# Association between English Proficiency and Kidney Disease Knowledge and Communication Quality among Patients with ESKD

**DOI:** 10.34067/KID.0000000000000398

**Published:** 2024-02-15

**Authors:** Ashley Martinez, Austin Warner, Neil R. Powe, Alicia Fernandez, Delphine S. Tuot

**Affiliations:** 1 Division of Nephrology, Stanford University School of Medicine, Stanford, California; 2 Loyola University Chicago Stritch School of Medicine, Maywood, Illinois; 3 Department of Medicine, San Francisco School of Medicine, University of California, San Francisco, San Francisco, California; 4 Zuckerberg San Francisco General Hospital and Trauma Center, San Francisco, California; 5 Center for Vulnerable Populations, San Francisco School of Medicine, University of California, San Francisco, San Francisco, California; 6 Division of Nephrology, University of California, San Francisco, San Francisco, California

**Keywords:** chronic dialysis, patient satisfaction, patient-centered care

## Abstract

**Key Points:**

- In one hospital-based safety-net dialysis unit, only one half of patients with ESKD knew their cause of kidney failure, which did not differ by English proficiency status.
- Patients with limited English proficiency (versus English-proficient patients) reported poorer communication with the dialysis care team (less listening, fewer clear explanations, less time spent).
- We highlight the need for tailored, patient-centered communication between limited English-proficient patients and dialysis care team members.

**Background:**

ESKD is a chronic health condition for which communication between health care teams and patients is important to guide patient self-management activities. Yet, little is known about the quality of communication among patients with ESKD and their care team members. We examined the influence of patient's limited English proficiency (LEP) status on communication experiences at one dialysis center.

**Methods:**

A survey was administered to adults receiving ESKD care at a dialysis unit within a public health care delivery system between July 2022 and February 2023, to ascertain kidney disease knowledge and perceptions of communication quality with the dialysis care team. Multivariable logistic and ordinal logistic regression models adjusted for age and sex were used to determine associations between LEP status and CKD knowledge.

**Results:**

Among 93 eligible patients, 88.2% (*n*=82) completed the survey. Approximately 37.8% (*n*=31) had LEP, mean age was 58.8 years, 68.3% were men, mean dialysis vintage was 3.9 years, and 25% had a positive depression screen (LEP 30%; English-proficient 22%). A higher proportion of English-proficient patients screened positive for limited health literacy compared to those with LEP (74.5% versus 38.7%, *P* = 0.002). Overall, knowledge of assigned cause of ESKD (53.4%) and CKD/transplant knowledge (57.3%) was suboptimal. After adjustment, LEP status was not significantly associated with knowing the correct cause of kidney failure (odds ratio, 0.49; 95% confidence interval, 0.19 to 1.27) but was significantly associated with having a higher score on a CKD/transplant knowledge scale (odds ratio, 3.99; 95% confidence interval, 1.66 to 9.58). Patients with LEP reported poorer communication quality with dialysis providers and staff (less listening, fewer clear explanations, less time spent with patients) compared with English-proficient patients, although differences were not statistically significant.

**Conclusions:**

Overall communication between patients with ESKD and members of the dialysis care team was suboptimal, regardless of English proficiency. Interventions to enhance communication for ESKD patients are needed.

## Visual Abstract

**Figure:**
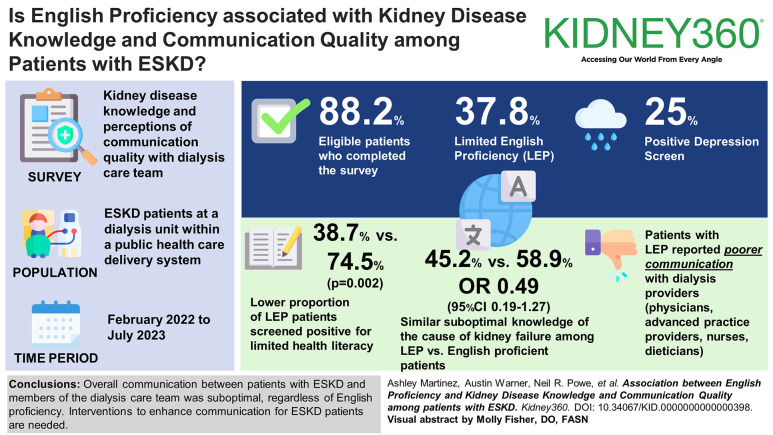

## Introduction

The number of people in the United States who report speaking a language other than English at home has risen dramatically in the past few decades. In 1980, this number was approximately 23 million people, and in 2019, this number had grown to 68 million people, a 194% increase. Spanish is the most commonly reported non-English language spoken at home, followed by Chinese languages. Of individuals 5 years and older who report speaking a language other than English at home, 37.6% of those individuals have limited English proficiency (LEP),^1^ including 38.6% of Spanish speakers and 48% of speakers of Chinese languages.^1,2^ The LEP patient population in the United States is a heterogeneous group, with differing countries of origin, culture, ethnicity, and sociodemographic factors.^1–3^ However, they share the common experience of having to navigate language barriers in a multitude of settings, including health care.^4–6^

Among adults, LEP status is associated with worse outcomes for individuals with chronic health conditions such as diabetes and heart failure.^7–10^ Although multifactorial, one likely contributor is suboptimal communication about those conditions between LEP patients and their provider teams.^11–14^ There are also well-documented disparities in access to care, adherence to preventive services, and receipt of high quality primary care by LEP status.^15–17^ Several studies have documented improvement of health outcomes with the provision of language concordant care, underlining the importance of addressing language barriers for patients with LEP.^18–21^ For example, in one study by Parker *et al.* the authors found significant improvements in hemoglobin A1c and LDL cholesterol levels for a sample of LEP patients with type 2 diabetes after the transfer of their care from a language-discordant to a language-concordant provider.^20^

ESKD is a chronic health condition for which English proficiency may matter. Patients with ESKD are required to perform active self-management with guidance from the dialysis team, including consuming low potassium, low phosphorous diets; restricting fluid intake; adhering to complicated medication regimens; and participating in physical activity to maintain sufficient stamina for a future kidney transplant surgery.^22–24^ In addition, patient knowledge about the etiology of their kidney failure may inform patient and family member decisions about kidney transplantation and living donation. Among patients with ESKD, inaccurate self-reporting of comorbid conditions has been associated with increased all-cause mortality, further highlighting the importance of patient–provider communication on disease management.^25^ This is similar to previous studies about other chronic health conditions that have shown that increasing patient education, understanding, and awareness results in positive effects on health outcomes.^26–28^ Yet, despite the need for frequent communication between patients with ESKD and their providers, quality of communication between patients with ESKD and members of their health care team has not been investigated in depth. Research into this area is important because communication is one factor of ESKD care delivery that may be modifiable; for individuals with LEP, this could include greater use of professional interpreters, multilingual staff and clinicians, and language concordant health-related materials. We sought to fill this gap in knowledge by examining the association between LEP and communication experiences among patients with ESKD receiving dialysis treatments at a dialysis center within a public health care delivery system.

## Methods

### Study Design, Setting, and Participants

This was a cross-sectional survey study. Eligible patients included adults age 18 years and older who received renal replacement therapy with either hemodialysis or peritoneal dialysis at one safety net outpatient dialysis center between July 21, 2022, and February 1, 2023. Patients with dementia were excluded from the study, as were individuals who could not communicate verbally or who were deaf.

### Consent Process and Survey Administration

Patients receiving hemodialysis were recruited in person during a treatment session while those receiving peritoneal dialysis were approached either during an in-person clinic visit or *via* telephone call. Informed consent was obtained, leveraging translated consent forms in the patient's primary language and professional telephone interpreters. After informed consent, study participants had the option to complete the survey immediately or at a future date if they expressed fatigue during the consent process. Surveys were administered by study staff either in person or *via* telephone. Survey answers were simultaneously entered into a Health Insurance Portability and Accountability Act-compliant electronic database, Research Electronic Data Capture^29^ by a study team member. The study was approved by the University of California, San Francisco institutional review board (22-36322).

### Primary Predictor

The primary predictor was LEP status. LEP was defined as requiring an interpreter for the survey and responding less than “well” when posed the US Census Bureau survey question: How well do you speak English?, (answer choice options were as follows: “not at all,” “not well,” “well,” and “very well”).^30^

### Outcomes

The main outcome was patient knowledge of the assigned cause of their kidney failure, ascertained by whether patient survey responses matched those documented by the nephrologist on the 2728 Medical Evidence of ESKD Form. A second comprehension outcome was CKD and transplant knowledge ascertained by a CKD/transplant comprehension scale comprising three selected true/false questions from the validated Knowledge Assessment of Renal Transplantation 2.0 questionnaire: (*1*) Kidney disease increases a person's chance of a heart attack; (*2*) in general, patients can live longer with a kidney transplant than if they stayed on dialysis; and (*3*) in general, most people on dialysis are happier with the quality of their lives than people with transplants.^31^ From these questions, a four-category ordinal scale was developed, ranging from 0 (the patient had answered 0/3 questions correctly) to 3 (the patient had answered 3/3 questions correctly). Other outcome measures included patients' perceptions of the helpfulness and supportiveness of staff at the dialysis unit including the frequency of attentive listening, provision of clear explanations, and adequacy of time spent by dialysis team members and patient self-report of conversations occurring with providers about kidney-friendly dietary practices, home dialysis options, and transplant candidacy.

### Measurements

Sociodemographic data were self-reported, including information on race/ethnicity, gender identity, English proficiency and languages spoken, social support, and socioeconomic status. Participants were screened for depression using the patient health questionnaire-2. A cutoff score ≥3 is considered as a positive screen for depression in the United States.^32^ Health literacy was ascertained by the validated question, how confident are you filling out medical forms by yourself?^33^ Patient age, dialysis vintage, and transplant referral status were obtained from chart review. The assigned cause of kidney failure was collected from the 2728 Medical Evidence of ESKD Form.

### Statistical Analysis

To assess for sociodemographic differences between patients with and without LEP, Fischer exact tests and *t* tests of association were performed. Univariate associations between LEP status and outcomes were ascertained by Fischer exact tests and rank sum tests. Multivariable logistic regression adjusted for age and sex was used to estimate the presence, direction, strength, and independence of an association between English proficiency status and patient's correct identification of their ESKD cause, our main outcome of interest. For the secondary comprehension outcome, an ordinal logistic regression model also adjusted for age and sex was used to assess CKD/transplant knowledge among patients with LEP compared with those with English proficiency. An odds ratio (OR) >1.0 indicated greater knowledge on the ordinal CKD/transplant comprehension scale. Statistical analyses were performed using Stata/SE, version 17.0 (StataCorp).

## Results

### Characteristics of the Study Sample

Among 93 eligible patients, 82 individuals completed the survey, for an 88.2% response rate. The patient population was diverse (39% Hispanic, 22% Asian, 19.5% Black, 7.3% non-Hispanic White, 6.1% Native Hawaiian or Pacific Islander, and 6.1% other race patients) and 37.8% (*n*=31) self-reported having LEP (Table 1). Among those with LEP, Spanish was the most frequently spoken language (77.4%), followed by Cantonese (12.9%) and Tagalog (9.7%). The mean age was 58.8±13.4 years, 68.3% were men, and the mean dialysis vintage was 3.9±3.8 years, without significant differences by LEP status. Compared with English-proficient patients, a greater proportion of patients with LEP reported less than a high school level of education, yet though those with English proficiency were more likely to screen positive for limited health literacy than their LEP counterparts (74.5% versus 38.7% of patients, respectively, *P* = 0.002).

**Table 1:** Sample characteristics of dialysis patients by English proficiency status

Self-Reported English Proficiency	All (*N*=82)^a^	LEP (*n*=31)^b^	English Proficient (*n*=51)^c^	*P* Value^d^
Age, yr, mean±SD	58.8±13.4	56.6±16.3	60.1±11.2	0.25
Male sex, *No.* (%)	56 (68.3)	21 (67.7)	35 (68.6)	1.0
**Race/ethnicity, *No.* (%)**				
Hispanic	32 (39)	24 (77.4)	8 (15.7)	
Black, non-Hispanic	16 (19.5)	0 (0)	16 (31.4)	
Asian	18 (22)	7 (22.6)	11 (21.6)	
Native Hawaiian or Pacific Islander	5 (6.1)	0 (0)	5 (9.8)	
White, non-Hispanic	6 (7.3)	0 (0)	6 (11.8)	
Other	5 (6.1)	0 (0)	5 (9.8)	<0.001
**Primary language if LEP, *No.* (%)**				
Spanish	24 (29.3)	24 (77.4)	N/A	
Cantonese	4 (4.9)	4 (12.9)	N/A	
Tagalog	3 (3.7)	3 (9.7)	N/A	
Limited health literacy^e^, *No.* (%)	50 (60.1)	12 (38.7)	38 (74.5)	0.002
**Education, *No.* (%)**				
<High school	35 (43.2)	16 (51.6)	19 (38.0)	
Completed high school	24 (29.6)	11 (35.5)	13 (26.0)	
Associate degree/some college	12 (14.8)	2 (6.5)	10 (20.0)	
Completed college or more	10 (12.4)	2 (6.5)	8 (16.0)	0.18
**Annual household income, *No.* (%)**				
<$10,000	18 (22.5)	4 (12.9)	14 (28.6)	
$10,000–$19,999	22 (27.5)	7 (22.6)	15 (30.6)	
$20,000–$49,999	7 (8.8)	2 (6.5)	5 (10.2)	
Patient states, “I don't know”	33 (41.3)	18 (58.1)	15 (30.6)	0.11
At risk for food insecurity^f^, *No.* (%)	23 (29.1)	15 (50.0)	8 (16.3)	0.002
**Dialysis modality, *No.* (%)**				
Hemodialysis	64 (78)	21 (67.7)	43 (84.3)	
Peritoneal dialysis	18 (22)	10 (32.3)	8 (15.7)	0.10
Years on dialysis, mean±SD	3.9±3.8	4.7±4.0	3.4±3.7	0.16

LEP, limited English proficiency; N/A, not applicable.

a *n*=82 for all rows except: education (*n*=81) and at risk for food insecurity (*n*=79).

b *n*=31 for all rows analyzing participants with limited English proficiency except: at risk for food insecurity (*n*=30).

c *n*=51 for all rows analyzing participants with English proficiency except: education (*n*=50); annual household income (*n*=49); at risk for food insecurity (*n*=49).

d Fisher exact test used for all rows except: age and years on dialysis (which used *t* tests)

e Limited health literacy identified by use of the single-question screening tool, how confident are you filling out medical forms by yourself?

f At risk for food insecurity defined as a response of yes to the question, in the last 12 months, did you ever eat less than you felt you should because there was no enough money for food?

Overall, few patients rated their general health as very good (9.8%) or excellent (12.2%). Similarly, few patients rated their quality of life as very good (17.1%) or excellent (12.2%). LEP status was associated with reporting less friend or family support compared with English-proficient patients (74.2% versus 84.3%, *P* = 0.02). A quarter of the patients (25%) screened positive for the likelihood of major depressive disorder, including 30% of those with LEP and 22% of those with English proficiency (Table 2).

**Table 2:** Measures of mood and well-being by English proficiency status

Self-Reported English Proficiency	All, *No.* (%) (*N*=82)^a^	LEP, *No.* (%) (*n*=31)^b^	English Proficient, *No.* (%) (*n*=51)^c^	*P* Value^d^
At risk for major depressive disorder^e^, *No.* (%)	20 (25)	9 (30)	11 (22)	0.44
**Has friend or family support^f^, *No.* (%)**				
Yes	66 (80.5)	23 (74.2)	43 (84.3)	
No	11 (13.4)	8 (25.8)	3 (5.9)	
Patient states “I do not want help”	4 (4.9)	—	4 (7.8)	
Patient states “I don't know”	1 (1.2)	—	2 (2)	0.02
**Self-rating of overall health^g^, *No.* (%)**				
Excellent	10 (12.2)	5 (16.1)	5 (9.8)	
Very good	8 (9.8)	2 (6.5)	6 (11.8)	
Good	31 (37.8)	9 (29)	22 (43.1)	
Fair	26 (31.7)	15 (48.4)	11 (21.6)	
Poor	7 (8.5)	—	7 (13.7)	0.90
**Self-rating of quality of life^h^, *No.* (%)**				
Excellent	10 (12.2)	2 (6.5)	8 (15.7)	
Very good	14 (17.1)	5 (16.1)	9 (17.7)	
Good	28 (34.2)	12 (38.7)	16 (31.4)	
Fair	22 (26.8)	10 (32.3)	12 (23.5)	
Poor	8 (9.8)	2 (6.5)	6 (11.8)	0.51

LEP, limited English proficiency.

a *n*=82 for all rows except: patient health questionnaire-2 depression screen (*n*=80).

b *n*=31 for all rows analyzing participants with limited English proficiency except: patient health questionnaire-2 depression screen (*n*=30).

c *n*=51 for all rows analyzing participants with English proficiency except: patient health questionnaire-2 depression screen (*n*=50).

d Fisher exact test used for at risk for major depression and friend or family support, Rank sum test used for self-ratings of overall health and quality of life.

e At risk for major depressive disorder identified by scoring ≥3 points on the patient health questionnaire-2.

f Ascertained by the question, can you count on any friends or family to provide you with emotional support such as talking over problems or helping you make a difficult decision?

g Ascertained by the question, in general, how would you rate your overall health right now?

h Ascertained by the question, in general, how would you rate your overall quality of life?

### Patient Perceptions of Communication

**Figure 1:**
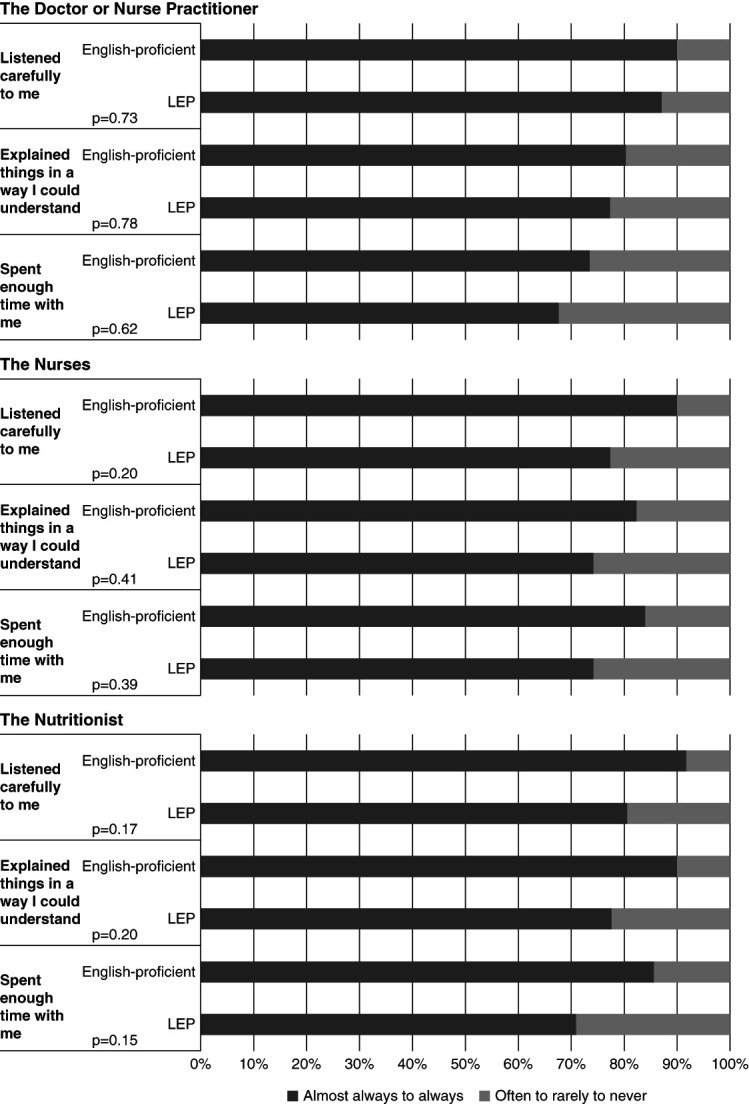
**Patient-perceived communication quality with dialysis care team members.** LEP, limited English proficiency.

**Figure 2:**
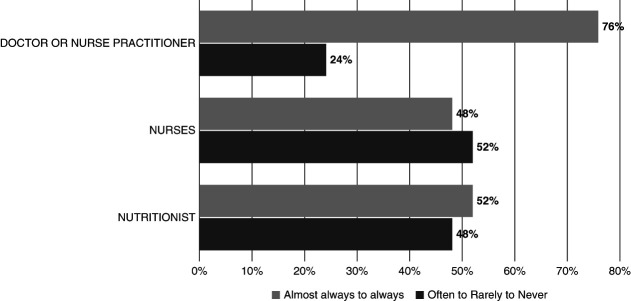
Patient-reported frequency of interpreter use by dialysis team member when a provider did not speak patient's primary language.

A high proportion of patients reported speaking with their nephrologist about kidney transplant referral, home dialysis modalities, and healthy dietary and lifestyle habits, without statistically significant differences by LEP status (Supplemental Table 1). However, in response to the question, how often did you feel staff… were as helpful as you thought they should be?, only 77.4% of LEP patients responded always or almost always compared with 89.8% of English-proficient patients (*P* = 0.20). When asked about specific interactions with care team members, LEP patients reported less listening, receiving fewer clear explanations, and staff members spending less time with them compared with English-proficient patients, although these differences were not statistically significant (Figure 1 and Supplemental Table 2). Among participants with LEP, only 76% stated that their physician always or almost always used a professional interpreter to communicate (Figure 2). Patient-reported use of professional interpreters was even lower for nutritionist encounters (52%) and nursing encounters (48%).

### Patient Knowledge

**Figure 3:**
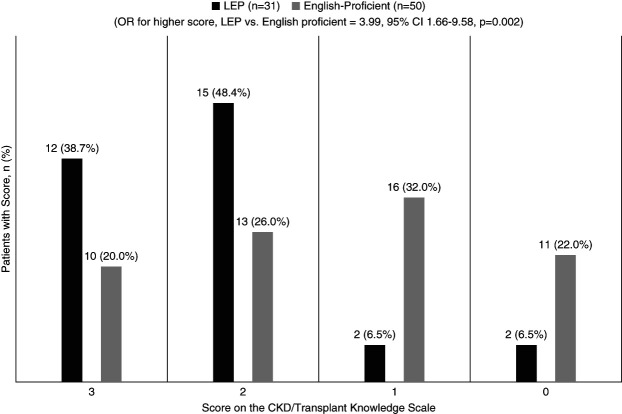
**Patient scores on the CKD/transplant knowledge scale by English proficiency status.** Distribution of the comprehension outcome for each ordered group by score: a score of 3 represents answering 3 of 3 questions correctly, a score of 2 represents answering 2 of 3 questions corrections, a score of 1 represents answering 1 of 3 questions correctly, and a score of 0 represents answering 0 of 3 questions correctly. CI, confidence interval; OR, odds ratio.

Approximately half of the patients (53.4%) correctly identified their assigned cause of ESKD, with fewer LEP patients compared with their English-proficient counterparts (45.2% versus 58.8%, *P* = 0.26). In analyses adjusted for patient age and sex, patients with LEP had lower, but not statistically significant, odds of knowing the assigned cause of kidney failure as compared with those with English proficiency (OR, 0.49; 95% confidence interval, 0.19 to 1.27). Among the overall study sample, patients answered an average of 57.3% of CKD and transplant knowledge questions correctly. When comparing English proficiency status, LEP patients answered an average of 73.1% of CKD and transplant knowledge questions correctly compared with 47.7% for English-proficient patients (*P* ≤ 0.001). After adjustment for age and sex, LEP status was associated with four-fold higher odds of having a higher score on an ordinal CKD/transplant knowledge scale as compared with those with English proficiency (OR, 3.99; 95% confidence interval, 1.66 to 9.58; Figure 3). Thus, despite poor patient perception of communication, patient CKD and transplant knowledge was found to be similar or higher among patients with LEP.

## Discussion

This study about LEP and communication in patients with ESKD has two main findings. First, regardless of English proficiency status, patient knowledge of the assigned cause of their kidney failure is poor. Second, overall communication among dialysis team members and patients with ESKD is suboptimal, and patient perception of communication quality trends toward poorer quality among LEP patients. If replicated in larger, multicenter studies, these findings have implications for how kidney care can be optimized to reduce known racial/ethnic inequities in adverse health outcomes.

Nearly one half of patients who participated in this study did not know what cause their nephrologist had assigned to their kidney failure. Understanding the cause of kidney failure has important scientific implications for the management of nonrenal manifestations of systemic disorders among individuals with ESKD (*i.e*., rash, altered mental status, poor cardiovascular health in individuals with kidney failure from systemic lupus erythematosus^34^) and the care and management of patients after a kidney transplant (*i.e*., monitoring of proteinuria for recurrence of kidney disease among individuals with idiopathic focal segmental glomerulosclerosis). It may also have important implications for genetic testing of family members of patients with ESKD. Suboptimal knowledge of cause of kidney failure in this study is aligned with national data that suggest that <50% of individuals with very advanced CKD are aware of their disease at all, much less the etiology. Although low CKD awareness among individuals with predialysis CKD has been attributed in part to poor communication about CKD between primary care providers and patients,^35^ our data suggest that conversations about cause of kidney failure may also be unsatisfactory between nephrologists and individuals with ESKD.

Given a low proportion of individuals in the study correctly reported their cause of kidney failure, it was surprising that general knowledge about CKD and transplant was higher. Perhaps this apparent discrepancy is because of more frequent communication between dialysis patients and their care teams about self-management strategies, including kidney transplant and healthy dietary and lifestyle habits, compared with etiology of kidney failure. Approximately 80% of individuals with ESKD reported discussing these topics with their providers, regardless of LEP status. These results could also be explained by the higher health literacy observed in the LEP patient population compared with their English-proficient counterparts. Low health literacy has been associated with increased mortality among individuals with ESKD requiring hemodialysis.^36^ Although speculative, low health literacy may confer a heavier impact on CKD and transplant knowledge compared with language barriers, especially given the availability of professional interpreters who can mitigate language barriers.

Another hypothesis to explain these data might also be a center-specific effect. Perhaps an attuned safety net health care system can attenuate the effects of LEP. The safety net dialysis unit where this study was conducted may have providers with enhanced skills for communicating with diverse patient populations or a center-specific increased commitment to ensuring patients with LEP are not adversely harmed because of language barriers. Ensuring the availability of robust language interpretation services would, of course, be in the best interest of a dialysis center that serves a large LEP population and financial incentives for dialysis units may further promote patient dialysis team communication efforts. For example, the Centers for Medicare and Medicaid Services' Kidney Care Choices Model incentivizes participating providers to report quality metrics, including patient activation measures.^37^ Patient activation requires that a patient is empowered through sufficient health knowledge and skills to be able to effectively plan and participate in their care and has been found to be strongly associated with patient–provider communication in the Latino patient population.^38^ Conferring sufficient health knowledge to the LEP patient likely requires effective language tools to ensure communication is adequate. Although patient reports of the use of professional interpreters by all dialysis team members was not 100% in this study, its availability and relatively high use among clinicians may have helped bridge the linguistic divide. Previous studies have demonstrated that presence of a robust interpretation system can enhance awareness and receipt of guideline-concordant CKD care among individuals with CKD and LEP.^39,40^ It is likely that the same is true among individuals with ESKD.

However, despite the availability of interpreter services, LEP study participant responses to communication quality survey questions trended toward dialysis care team members being less helpful, listening less, offering less clear explanations, and spending less time with them compared with patients with English proficiency, although differences were not statistically significant. These results may portend less patient satisfaction for patients with LEP and ESKD compared with those with English proficiency, which would be consistent with other studies which have examined LEP patient satisfaction. In a study by Rivadeneyra *et al.*, patient-centeredness scores were found to be lower for interactions with Spanish-speaking patients even with the presence of an interpreter as compared with -peaking patients, suggesting that Spanish-speaking patients may experience less satisfaction with language-discordant doctors even when communicating with the assistance of a professional interpreter.^41^

Language isolation has been linked to depressive symptoms in the elderly Latino population ^42^ and similarly may have contributed to the higher proportion of worrisome depression screens observed among LEP patients in our study. In 2020, the estimated prevalence of major depressive disorder was 8.4% among US adults. Data from one systematic review by Palmer *et al.* measured a much higher prevalence of 22.8% among adults with ESKD.^43^ We found a similar overall prevalence (25%), and an even higher risk among individuals with LEP (30%). Patients with LEP also reported less social support networks as compared with English-proficient patients, which may be an additional driver for increased depressive symptoms. One study by Grav *et al.* found that a lack of perceived emotional social support was associated with 3.14 times the odds of depression among adults.^44^ Helping patients with LEP find more social support and using language concordant collaborative care teams may be one avenue by which dialysis centers may address this important disparity. The collaborative care model that leverages a multidisciplinary team approach to engage and monitor patients has been demonstrated to be effective in depression management among LEP patients when using the use of bilingual team members.^45^

To our knowledge, this is one of the first studies among patients with ESKD to examine the relationship between language proficiency and patient understanding of kidney disease. The main study limitation was its small sample size, thus limiting the power to detect statistically significant differences between patients with and without LEP. Sample size calculations were not performed before study initiation given the paucity of data about communication among English-proficient versus limited English-proficient patients with ESKD. An additional limitation was that the dialysis unit in which study participants were recruited may be more linguistically diverse than many across the United States, which may have led to greater dialysis team awareness about language barriers and use of professional interpreters compared with other dialysis centers. We assume that study results may thus underestimate actual differences by LEP status relative to the other dialysis populations in the United States. Despite these limitations, we highlight multiple areas for improvement for the ESKD patient population with LEP including a need for superior communication quality and patient centeredness during patient–provider interactions and a need for tailored support for those experiencing depression and social isolation. Additional research for ESKD patients with LEP should be pursued because expanding data in this field are crucial to discovering impactful interventions for this vulnerable, growing patient population.

## Supplementary Material

## Data Availability

All data are included in the manuscript and/or supporting information.

## Supplemental Material

Supplemental Table 1. Topics of communication between patient and nephrologist by English proficiency status.

Supplemental Table 2. Patient-reported quality of interactions with specific dialysis team members by English proficiency status.
