# Supplementary material for: Association between English Proficiency and Kidney Disease Knowledge and Communication Quality among Patients with ESKD
Source: Kidney360. 2024 Feb 15;5(4):560–8. doi: 10.34067/KID.0000000000000398 (PMC11093550; doi:10.34067/KID.0000000000000398)
Supplement: Supplementary file 1 [file kidney360-5-560-s001.pdf]

Supplementary Material

Table S1. Topics of Communication between patient and nephrologist by English Proficiency Status

Self-reported English Proficiency	All n (%) (n = 82) †	LEP n (%) (n = 31) †	English Proficient n (%) (n = 51) ‡	p-value for the Fisher's exact test
**Patients who knew whether they had been referred for transplant evaluation**	65 (79.3)	26 (83.9)	39 (76.5)	0.58
**Patients who reported their nephrologist had discussed home methods of dialysis** *	42 (65.6)	15 (71.4)	27 (62.8)	0.58
**Patients who reported their nephrologist had discussed…**				
Limiting dietary salt intake	68 (84)	25 (80.7)	43 (86.0)	0.55
Limiting fluid intake	69 (85.2)	25 (80.6)	44 (88.0)	0.52
Limiting dietary potassium intake *	54 (87.1)	18 (85.7)	36 (87.8)	1.00
Physical activity	63 (76.8)	25 (80.7)	38 (74.5)	0.60

LEP = Limited English Proficiency

* Responses elicited only from patients receiving hemodialysis method of dialysis therapy

† n = 82 for all rows except: Patients who reported their nephrologist had discussed home methods of dialysis (n = 64); Limiting dietary salt intake (n = 81); Limiting fluid intake (n = 81); Limiting dietary potassium intake (n = 62).

‡ n = 31 for all rows analyzing participants with LEP except: Patients who reported their nephrologist had discussed home methods of dialysis (n = 21); Limiting dietary potassium intake (n = 21).

§ n = 51 for all rows analyzing participants with English-proficiency except: Patients who reported their nephrologist had discussed home methods of dialysis (n = 43); Limiting dietary salt intake (n = 50); Limiting fluid intake (n = 50); Limiting dietary potassium intake (n = 41).

Table S2. Patient reported quality of interactions with specific dialysis team members by English Proficiency Status

Self-reported English Proficiency	All (n = 82) *	LEP (n = 31)	English Proficient † (n = 51)	p-value for the Fisher's exact test
**Patients felt the doctor or nurse practitioner… n (%)**				
**Listened carefully**	72 (88.9)	27 (87.1)	45 (90.0)	
Almost always to Always	9 (11.1)	4 (12.9)	5 (10.0)	0.73
Often to Rarely to Never				
**Explained things clearly**	65 (79.3)	24 (77.4)	41 (80.4)	
Almost always to Always	17 (20.7)	7 (22.6)	10 (19.6)	0.78
Often to Rarely to Never				
**Spent enough time with them**	57 (71.3)	21 (67.7)	36 (73.5)	
Almost always to Always	23 (28.8)	10 (32.3)	13 (26.5)	0.62
Often to Rarely to Never				
**Patients felt the nurses… n (%)**				
**Listened carefully**				
Almost always to Always	69 (85.2)	24 (77.4)	45 (90.0)	
Often to Rarely to Never	12 (14.8)	7 (22.6)	5 (10.0)	0.20
**Explained things clearly**				
Almost always to Always	65 (79.3)	23 (74.2)	42 (82.4)	
Often to Rarely to Never	17 (20.7)	8 (25.8)	9 (17.7)	0.41
**Spent enough time with them**				
Almost always to Always	65 (80.3)	23 (74.2)	42 (84.0)	
Often to Rarely to Never	16 (19.8)	8 (25.8)	8 (16.0)	0.39
**Patients felt the nutritionist… n (%)**				
**Listened carefully**				
Almost always to Always	70 (87.5)	25 (80.7)	45 (91.8)	
Often to Rarely to Never	10 (12.5)	6 (19.4)	4 (8.2)	0.17
**Explained things clearly**				
Almost always to Always	69 (85.2)	24 (77.4)	45 (90.0)	
Often to Rarely to Never	12 (14.8)	7 (22.3)	5 (10.0)	0.20
**Spent enough time with them**				
Almost always to Always	64 (80)	22 (70.8)	42 (85.7)	
Often to Rarely to Never	16 (20)	9 (29.0)	7 (14.3)	0.15

LEP = Limited English Proficiency

* n = 82 for all rows except: patients felt the doctor listened carefully (n = 81); patients felt the doctor spent enough time with them (n = 80); patients felt the nurses listened carefully (n = 81); patients felt the nurses spent enough time with them (n = 81); patients felt the nutritionist listened carefully (n = 80); patients felt the nutritionist explained thing clearly (n = 81); patients felt the nutritionist spent enough time with them (n = 80).

† n = 51 for all rows analyzing participants with English-proficiency except: patients felt the doctor listened carefully (n = 50); patients felt the doctor spent enough time with them (n = 49); patients felt the nurses listened carefully (n = 50); patients felt the nurses spent enough time with them (n = 50); patients felt the nutritionist listened carefully (n = 49); patients felt the nutritionist explained thing clearly (n = 50); patients felt the nutritionist spent enough time with them (n = 49).
